# Pharmacodynamic Biomarkers for Emerging LRRK2 Therapeutics

**DOI:** 10.3389/fnins.2020.00807

**Published:** 2020-08-06

**Authors:** Kaela Kelly, Andrew B. West

**Affiliations:** Duke Center for Neurodegeneration Research, Departments of Pharmacology and Cancer Biology, Neurology, and Neurobiology, Duke University, Durham, NC, United States

**Keywords:** LRRK2, LRRK2 kinase inhibitor, biomarker, pharmacodynamic markers, exosomes, Parkinson’s disease

## Abstract

Genetic studies have identified variants in the *LRRK2* gene as important components of Parkinson’s disease (PD) pathobiology. Biochemical and emergent biomarker studies have coalesced around LRRK2 hyperactivation in disease. Therapeutics that diminish LRRK2 activity, either with small molecule kinase inhibitors or anti-sense oligonucleotides, have recently advanced to the clinic. Historically, there have been few successes in the development of therapies that might slow or halt the progression of neurodegenerative diseases. Over the past few decades of biomedical research, retrospective analyses suggest the broad integration of informative biomarkers early in development tends to distinguish successful pipelines from those that fail early. Herein, we discuss the biomarker regulatory process, emerging LRRK2 biomarker candidates, assays, underlying biomarker biology, and clinical integration.

## Introduction

Neurological disorders, including neurodegenerative diseases, were among the areas with the lowest probability of new compound success over the 2010–2017 time period, with lack of efficacy being the primary cause of attrition (Morgan et al., 2012; Dowden and Munro, 2019). Further, many genes and processes associated with neurodegenerative diseases are not considered traditional parts of the so-called druggable proteome associated with clinically efficacious drugs (Hopkins and Groom, 2002). More than 800 Food and Drug Administration (FDA) approved small molecules and biotech drugs typically fall into predictable classes of proteins and enzymes that compose the known druggable proteome, and few of these known druggable targets are clearly linked to neurodegenerative disease (Wishart et al., 2006). However, with the identification of missense mutations in *LRRK2*, a new drug target emerged (Zimprich et al., 2004; West et al., 2005; Healy et al., 2008). *LRRK2* encodes protein kinase and GTPase domains, similar to domains present in some proteins within the druggable proteome. While the exact mechanisms of mutant LRRK2-induced neurodegeneration remain elusive and are not the focus of this review, the “kinase-activation” hypothesis for LRRK2-linked disease has advanced forward to novel therapeutic approaches (West et al., 2005; West, 2015, 2017). Recently, small molecule inhibitors and anti-sense oligonucleotides have progressed into clinical trials (e.g., clinicaltrials.gov NCT03976349, NCT04056689).

While it is standard practice to collect extensive pharmacokinetic (PK) data for drugs in early clinical efforts, these data poorly predict proof of mechanism (Morgan et al., 2018). PK measures typically define drug properties related to absorption, distribution, metabolism, and excretion (ADME). Convincing pharmacodynamic measures that would otherwise assess relationships between drug concentration at the site of action (e.g., receptor binding) and the resulting biochemical and physiological effects (e.g., enzyme activity) are not typically integrated into clinical trials for Parkinson’s disease. The lack of demonstrable efficacy in a clinical trial can be attributed to many factors, but insufficient biomarkers of target engagement and improper patient selection for clinical trials are often cited as primary causes (Morgan et al., 2012; Lopes et al., 2015; Smietana et al., 2016; Dowden and Munro, 2019). Long-duration clinical trials that seek to modify disease progression may be particularly susceptible to failures caused by a lack of biomarker integration.

As trials of investigational compounds targeting LRRK2 move forward, the apparent need for validated LRRK2-targeted biomarkers increases. Currently there are no biomarkers approved by the FDA that relate to LRRK2 pathobiology or activity. The development of informative pharmacodynamic biomarkers involves substantial investment and are not currently a requirement for the advancement of therapies in regulatory pipelines. Yet, as will be discussed herein, biomarker development may be key for successful clinical outcomes. In this review, we provide a rationale for prioritizing LRRK2-relevant biomarker development, an overview of possible integration in the development pipeline, and describe promising emergent candidates that measure different features related to LRRK2 pathobiology. While there is no clear single biomarker or approach that will shepherd LRRK2-targeted therapies through clinical trials, purpose-built panels with high evidentiary standards for meaningful measures in diverse populations may provide the best chance of identifying successful therapies.

## Importance of Biomarkers in Therapy Development

In 1998, the National Institutes of Health Biomarkers Definitions Working Group defined a biomarker as “a characteristic that is objectively measured and evaluated as an indicator of normal biological processes, pathogenic processes, or pharmacologic responses to a therapeutic intervention” (Biomarkers Definitions Working Group, 2001). According to the World Health Organization (WHO), measured responses may be functional and physiological, biochemical at the cellular level, or specify a molecular interaction. Biological markers that identify and monitor the biochemical effects of drugs may be theragnostic biomarkers that evaluate specific effects of a drug (e.g., target engagement) and downstream effects on pathogenic mechanisms. Theragnostic biomarkers may have practical utility in predicting positive outcomes. As a nascent target, there are currently no standard LRRK2-targeted biomarkers established for related drug development programs and clinical trials. However, as will be discussed, rapid advancements in the field position LRRK2 as a prime candidate for biomarker-based enrichment strategies in development pipelines.

**FIGURE 1:**
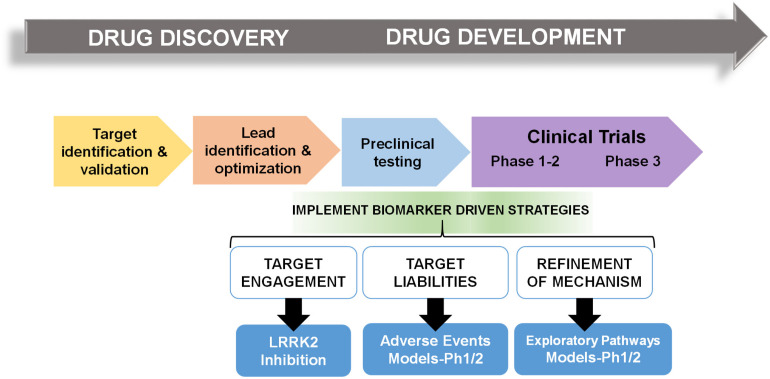
Biomarker positioning within the drug development pipeline. Preclinical studies may be used to refine and validate biomarkers in three broad categories, target engagement, liabilities, and pathways. Early implementation in phase I/II clinical trials may increase likelihood of success in efficacy trials.

In multiple retrospective analyses from large pharmaceutical companies, biomarker driven approaches have been identified as the most common difference between failed and successful efforts. Pfizer conducted an after-action review of 44 programs that reached a decision point in Phase II clinical trial between 2005 and 2009 and found that only 32% were deemed positive at their clinical proof-of-concept meeting (Morgan et al., 2012). Deeper analysis concluded that the programs with positive outcomes evaluated mechanism of action in humans, through biochemical biomarker classification of disease, typically with some evidence of target engagement. In contrast, nearly all terminated programs failed to adequately test mechanism. Pfizer’s findings prompted design guidelines for future projects using fundamental data and knowledge they termed the “three Pillars of survival” (Morgan et al., 2012). By these new standards, compounds must demonstrate (1) sufficient drug exposure at the target site over time, (2) target engagement, and (3) functional modulation of the target in order to advance to clinical development. Similarly, AstraZeneca sought to revise their research and development enterprise through new guidelines termed the “5R framework,” where three of the five “R” criterion require clear empirically derived and dynamic biomarker feedback (Morgan et al., 2018). Since implementing this revised strategy, project success rates across all stages of development improved for the 2012–2016 period compared to the 2005–2010 period, and at clinical proof-of-concept meetings, transition from candidate drug nomination to phase III completion improved by 19%. Furthermore, industry-wide surveys show that clinical trials that use biomarkers have higher overall success probabilities than trials without biomarkers (Wong et al., 2019). In an earlier study of 1,079 oncology drugs, success rates for drugs developed with biomarkers was 24 versus 6% for compounds developed without biomarkers (Lopes et al., 2015). Figure 1 illustrates points of biomarker integration in traditional drug discovery pipelines.

## Process for Biomarker Qualification

Numerous hurdles exist for transitioning novel biomarkers from the laboratory into clinical practice. There are numerous regulatory programs that facilitate the review and qualification of novel biomarkers for drug development (Amur et al., 2015). In 2004, the FDA introduced the Critical Path Initiative with hopes to improve the drug development process, where the advancement of new biomarkers was identified as a critical priority (Woodcock and Woosley, 2008). Furthermore, the FDA has clearly articulated necessary biomarker qualification standards. In alliance with the Critical Path Initiative, a systematic framework for developing evidentiary standards for biomarker qualification was developed by Pharmaceutical Research and Manufacturers of America (PhRMA) in partnership with the FDA and academia (Altar et al., 2008). In summary, the proposed context of use for a biomarker determines the level of evidence required to support qualification based on the tolerability of risk imposed. However, as might be expected with the paucity of biomarkers currently approved and in use in the clinic, evidentiary standards are not well defined for all types of biomarkers and their various context of uses.

**FIGURE 2:**
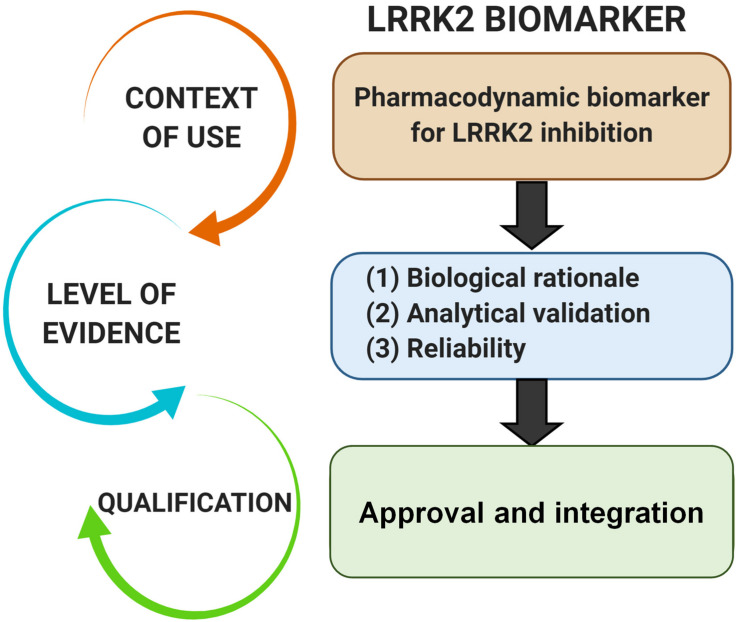
Evidentiary standards for qualification of LRRK2-targeted biomarkers depend on the intended context of use. In general, the intended use of a biomarker in drug development programs dictates the level of risk (e.g., impact of decision based on biomarker result) and engagement of regulators. Biomarkers utilized in making decisions in the clinic, or utilized in multiple programs, require higher levels of evidence and qualification.

Pharmacodynamic biomarkers are generally thought to be considered low risk because they are utilized early in drug development (e.g., exploratory) and are not typically decisional. Nascent biomarkers are typically used without regulatory qualification, but as pharmacodynamic biomarkers for target engagement evolve and enter the clinical space, greater evidentiary standards will be imposed. Factors that may contribute to evidentiary considerations for LRRK2-targeted biomarkers for target engagement include biological rationale, analytically validated methods, and reproducibility of data (Figure 2). As the different biomarkers advance in this pipeline, data will feedback over time to refine interpretations of context of use and biological rationale. We have argued in the past that a better understanding of the causal pathway for LRRK2 in PD pathogenesis will be critical for qualifying different biomarkers used to measure target and pathway engagement (West, 2015, 2017). Additionally, biomarker assays require high levels of specificity and sensitivity. As will be discussed, analytical methods for LRRK2-targeted biomarkers will need to be well established, with a foundational understanding of biological and technical variability. Both evidentiary factors, biological rationale, and validated assays, will first rely on technical reproducibility. In addition to test datasets, positive results in confirmatory datasets should provide the necessary level of evidence to support LRRK2-targeted biomarker qualification by regulatory bodies that include the FDA. Similar evidentiary frameworks exist in European Union guidelines (Biomarkers Definitions Working Group, 2001; Goodsaid and Papaluca, 2010).

Usage of the same FDA approved biomarkers across different studies may expedite the identification of successful LRRK2-targeted therapies. Of note, to establish a biomarker for the use in multiple development programs, a pharmaceutical developer, disease-specific foundation, health research organization, or consortium, must request regulatory qualification of a biomarker through the FDA Biomarker Qualification Program. This application process is distinct from the approval process for biomarker use in a single drug development program (e.g., one sponsor), where acceptance occurs through an Investigational New Drug (IND) application during the drug approval process. A review team is then assembled for the consultation and advice stage where preliminary data and analysis plans are evaluated. Once a biomarker has been qualified, it may then be used for its specified use of context within drug development programs. While there may be little pressure for individual developers to conform to standardization that might expedite the field as a whole, health-research funding bodies and foundations could reasonably insist, especially in pre-competitive phases of development, on utilization of standardized approved biomarkers.

## Emerging LRRK2 Biochemical Biomarkers

**FIGURE 3:**
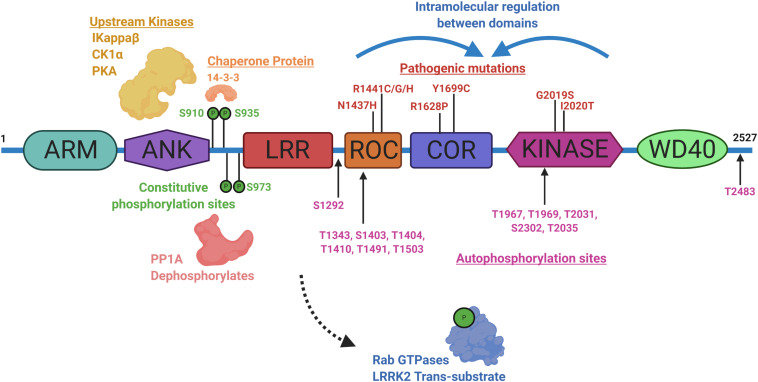
Illustration aligning LRRK2-conserved domain structure with constitutive phosphorylation sites (green), autophosphorylation sites (magenta), with pathogenic mutations (red). Kinases and phosphatases that can control constitutive phosphorylation are indicated together with 14-3-3 s. Intramolecular shifts in the ROC-COR-Kinase enzymatic stretch of domains may regulate LRRK2 activity and metabolism. ARM is armadillo-like, ANK is ankyrin-like, LRR is leucine-rich repeat, ROC is Ras-of-Complex Rab-like GTPase, COR is conserved C-terminal of ROC, Kinase is Ser/Thr-kinase domain, and WD40 is beta-transducin-like repeat.

Identifying physiological substrates of LRRK2 that correlate with LRRK2 kinase activity has been a priority goal since the discovery of mutations that biochemically upregulate LRRK2 kinase activity (West et al., 2005). Figure 3 highlights known LRRK2 phosphorylation sites and other protein regulators that serve as the basis for most LRRK2-targeted biomarker approaches. Direct measures of LRRK2 kinase activity in different cells and tissues became possible with the discovery of LRRK2 autophosphorylation at the Ser1292 residue, the most abundant LRRK2 autophosphorylation site near the Rab-like ROC domain within the LRRK2 protein (Sheng et al., 2012). Up to 30% of LRRK2 protein becomes phosphorylated at this residue *in vitro*, with much higher ratios observed in different biofluids (Wang et al., 2017). Biomarkers measuring autophosphorylated residues in several receptor-tyrosine kinases in different indications have been utilized in past clinical research (Paweletz et al., 2011; Wu et al., 2014; Zhang et al., 2016). LRRK2 also autophosphorylates several other threonine residues directly in the ROC domain *in vitro* (Greggio et al., 2009; Pungaliya et al., 2010; Liu et al., 2016), although these phosphorylated peptides have been more difficult to measure directly in cells and tissues, presumably due to their very low abundance, possibly less than 1% of the total pool of LRRK2 protein (Greggio et al., 2009; Gloeckner et al., 2010; Webber et al., 2011). This low-level of phosphorylation challenges current mass spectrometry-based sensitivities and antibody-differential affinities in binding phospho-peptides versus non-phospho-peptides.

A subset of small Rab GTPases have been identified as *trans*-substrates for LRRK2 kinase activity (Steger et al., 2016), with Rab10 phosphorylated by LRRK2 at the Thr73 residue (Eyers, 2018). The impact of pathogenic LRRK2 mutations on Rab10 phosphorylation are still under investigation, but with the administration of a LRRK2 inhibitor, pT73-Rab10 levels are reduced (Ito et al., 2016; Thirstrup et al., 2017). Dozens of other LRRK2 candidate substrates have been proposed, although a lack of evidence for LRRK2 phosphorylation under physiological conditions prevents broad integration in biomarker approaches (Pungaliya et al., 2010). LRRK2 was identified as a constitutively phosphorylated protein in a cluster of N-terminal residues including a serine residue at 935, although a kinase-inactivating mutation in LRRK2 did not ablate the levels of these phospho-sites (West et al., 2007). Curiously, small molecule inhibition more dramatically downregulates pS935 levels than kinase-inactivating mutations in LRRK2 (Dzamko et al., 2010). This regulation is suspected to be mediated within a cascade of 14-3-3 interaction and phosphatase activity that is affected by a conformational change in LRRK2 induced by inhibitor binding (Li et al., 2011; Dzamko et al., 2012; Sheng et al., 2012; Lobbestael et al., 2013; Liu et al., 2014; Kelly et al., 2018). Although an indirect measure, dephosphorylation of LRRK2 at Ser935 has been utilized extensively in development pipelines (Henderson et al., 2015; Perera et al., 2016; Thirstrup et al., 2017). As opposed to measures of phospho-Rab and pS1292-LRRK2, some LRRK2 pathogenic mutations, especially in the ROC domain (e.g., R1441C), appear to dramatically down-regulate pS935-LRRK2 levels (Delbroek et al., 2013; Muda et al., 2014; Giesert et al., 2017).

## LRRK2 Regulation in Biomarker Responses

**FIGURE 4:**
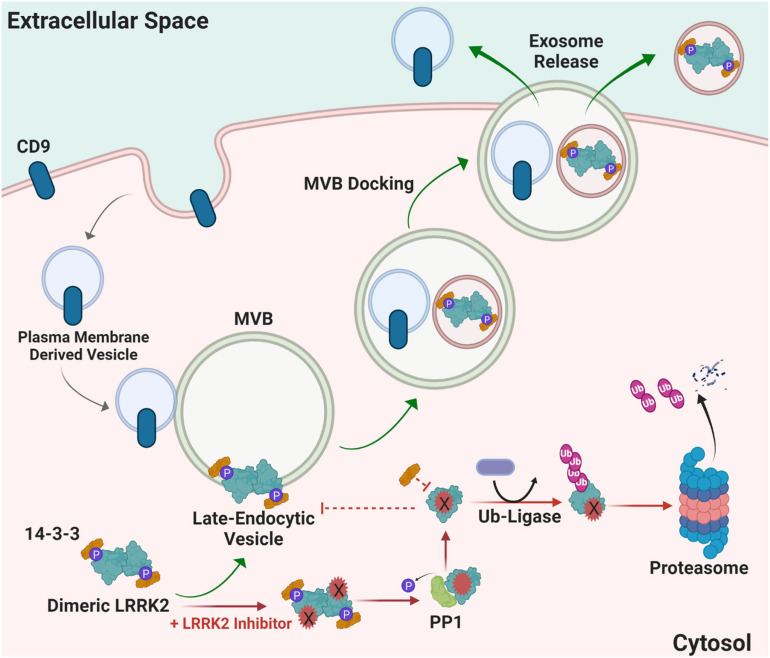
Proposed mechanism of how LRRK2 inhibition may alter LRRK2 turnover and protein-protein interactions necessary for localization and function. Distinct from canonical CD9-positive plasma membrane-derived vesicles, LRRK2 interacts with 14-3-3 proteins at multi-vesicular late-endosome vesicles. Intra-luminal budding of the endosome results in encapsulation inside of intralumenal vesicles that become exosomes when the endosome fuses with the plasma membrane. Alternatively, LRRK2 kinase inhibitors prevent 14-3-3 interactions and LRRK2 interaction with membranes, and instead favors ubiquitination and proteasome-dependent degradation. MVB multivesicular body, PP1 protein phosphatase 1, Ub ubiquitin, CD9 is CD9 Antigen; Leukocyte antigen MIC3.

LRRK2 functions in the endolysosomal pathway in both health and disease (Higashi et al., 2009; Tong et al., 2010; Piccoli et al., 2011; Matta et al., 2012). On a subcellular level, LRRK2 co-localizes with some membranes and vesicular structures, with apparent preference for mature-endosomes and lysosomes versus mitochondrial, nuclear, or endoplasmic reticulum (Biskup et al., 2006; Hatano et al., 2007; Alegre-Abarrategui et al., 2009; Dodson et al., 2012; Fraser et al., 2013). Little is known about how LRRK2 is regulated within the endocytic pathway and how LRRK2 therapeutics may affect these mechanisms long-term; however, it is hypothesized that LRRK2 inhibition may alter LRRK2 turnover and protein-protein interactions necessary for localization, function, and secretion in biofluids via exosome release (Figure 4). 14-3-3 chaperone proteins are highly expressed in the brain and have been implicated in the regulation of numerous neurodegenerative disorders including PD (Berg et al., 2003). 14-3-3 s interact with LRRK2, where binding is mediated by phosphorylation at residues Ser910 and Ser935 to alter LRRK2 subcellular localization (Dzamko et al., 2010; Nichols et al., 2010; Li et al., 2011; Lavalley et al., 2016). The 14-3-3 LRRK2 interaction may regulate LRRK2 association with late endosomes and uptake into multi-vesicular bodies (MVBs) with subsequent secretion of LRRK2 protein in exosomes (Fraser et al., 2013). CD9 is a ubiquitous transmembrane protein that traffics in plasma-membrane derived vesicles to MVBs during endocytosis and is often used a vesicular marker to identify an exosome’s source of origin (Willms et al., 2018). LRRK2 appears to be excluded from CD9-positive plasma-membrane endocytosed vesicles, suggesting that intraluminal budding events in the cytosol are the primary source for extracellular LRRK2, distinct from plasma membrane-derived exosomes (Fraser et al., 2013).

Finally, total LRRK2 protein levels, especially secreted LRRK2 in exosomes, may also be affected by LRRK2 inhibition. In many experimental observations, loss of LRRK2 kinase activity through inhibitor binding leads to decreased LRRK2 protein levels (Lobbestael et al., 2013, 2016; Fuji et al., 2015; Zhao et al., 2015; De Wit et al., 2019). Typically, enzymatic activity of autophosphorylating kinases are determined by the ratio of phosphorylated protein to total protein, for example pSer1292-LRRK2 or pSer935-LRRK2 to total LRRK2. Notable other examples include receptor-tyrosine kinases (Zhang et al., 2016). However, this phospho-to-total measure would be confounded in cases where total protein levels become low due to inhibitor treatment. A recent study evaluating LRRK2 inhibitors in non-human primate biofluids found that LRRK2 protein is unchanged in brain tissue but is diminished at varying levels in the periphery following acute treatment (Wang et al., 2020).

The effects of chronic LRRK2 inhibition on the endocytic pathway has yet to be fully understood. Studies using acute drug dosing strategies and kinase-dead LRRK2 mutants have provided evidence that the subcellular localization of LRRK2 is altered and reductions in kinase activity can lead to LRRK2 protein destabilization and degradation. A recent study in non-human primates showed that acute dosing with structurally distinct LRRK2 kinase inhibitors PFE-360 or MLi2 reduces total LRRK2 detection within exosomes isolated from urine and cerebral spinal fluid (CSF) (Wang et al., 2020). Additionally, there is evidence that LRRK2 kinase activity is crucial for maintaining steady-state levels of LRRK2. Mice expressing kinase-dead LRRK2 demonstrate markedly decreased levels of LRRK2 protein, and these results were mimicked pharmacologically (Herzig et al., 2011). LRRK2 inhibition and decreased phosphorylation of S935 has also been linked to increased ubiquitination. It has been proposed that after kinase inhibition occurs, phosphatases, such as PP1, are recruited to dephosphorylate LRRK2 and interrupt 14-3-3 binding, which then promotes the ubiquitination of LRRK2 and leads to proteasomal degradation (Zhao et al., 2015; Lobbestael et al., 2016). However, LRRK2 kinase inhibition *in vivo* does not always result in ubiquitous destabilization and degradation of LRRK2 (Daher et al., 2015; Fell et al., 2015; Henderson et al., 2015; Lobbestael et al., 2016; Kelly et al., 2018). Differential LRRK2 inhibition effects observed may relate to the specific compound, LRRK2 mutations, length of treatment, tissue, and/or cell types being evaluated.

## Emerging LRRK2 Biomarker Assays

LRRK2 biomarker candidates will require targeted quantitative assays for validation and clinical assay development. One challenge the field will face is developing a single assay that can reliably and accurately detect LRRK2 at varying levels in different biological matrices, and in formats that can be realistically implemented at clinical collection sites. While ELISA and PCR based assays represent the most common formats for approved biomarker tests, exosomal LRRK2 is considered a low-abundant protein source, and the concentration of LRRK2 in biofluids is usually at the low picomolar level below the limit of detection of many ELISA formats (Wang et al., 2019). Even with improved detection, immunodetection of peptides of interest (e.g., pSer1292-LRRK2) is dependent on the specificity and selectivity of the antibody in the given format. Further, throughput and ease of sample collection and preparation are priority variables for broad implementation. A high-throughput ELISA for pS935-LRRK2 levels in a 96-well format was developed to monitor the activity of endogenous LRRK2 in both rodent and human samples (Delbroek et al., 2013). An improved single-molecule based format from Quanterix has more recently been deployed to measure the abundant pS935-LRRK2 and total LRRK2 levels in human peripheral blood mononuclear cells (PBMCs) from PD patients and healthy controls (Padmanabhan et al., 2020). Single molecule-based and other ultrasensitive immunoassays for pSer1292-LRRK2 and LRRK2-Rab targets (e.g., pT73-Rab10) have not yet been described but hold tremendous promise.

**FIGURE 5:**
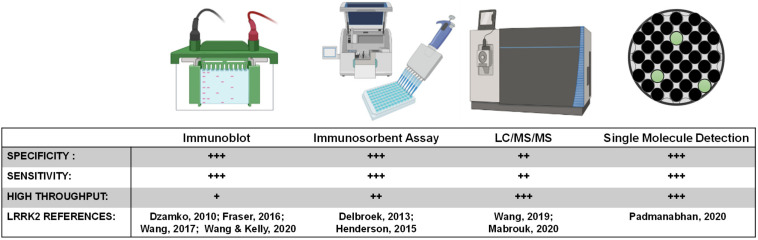
LRRK2 biomarker detection methods. Various methods exist for monitoring pharmacodynamic changes in LRRK2 kinase activity and protein levels. Implementing analytically validated biomarker assays for target engagement in early phase clinical trials will require high-throughput methods that are highly sensitive and specific for LRRK2.

Peptide detection via quantitative mass spectrometry has dramatically evolved in the last decade. While sensitivity may now rival single molecule-based immunoassays, the instruments are extremely expensive at present and complicated to run on a routine basis. Further, detection and quantification of single-phosphorylation events can be much more difficult than detection and quantification of total levels of protein that can utilize many peptides across the protein. Our past work measured pS1292-LRRK2 via mass spectrometry, notably requiring GluC protease digestion as opposed to canonical trypsin treatment (Wang et al., 2017). Although there are few approved biomarkers reliant on mass spectrometry detection, the next decade will certainly herald a new wave of antibody-agnostic assays for a variety of indications, possibly including LRRK2-targeted biomarkers. Or, mass spectrometry can be combined with efficacious antibodies. One promising approach for total LRRK2 protein measures in CSF uses a stable-isotope standard and capture by anti-peptide antibody approach (Mabrouk et al., 2020), and concentrations in CSF reported are very similar to those resolved by quantitative immunoblots (Wang et al., 2017). Figure 5 summarizes key biomarker development assays related to LRRK2 inhibition.

## Clinical Integration of LRRK2-Targeted Biomarkers

LRRK2 is not a ubiquitous protein but is expressed in many different cell types throughout the body. Neurons vulnerable to degeneration in PD all appear to express LRRK2 protein, as do immune cells responsive in disease, and represent the ostensible target LRRK2 protein for inhibition (West, 2017). Although expression in the brain is low, LRRK2 is abundantly expressed in immune cells, kidneys, and lungs (Biskup et al., 2007; Maekawa et al., 2010; Hakimi et al., 2011; Fuji et al., 2015). Tissue biopsy samples (e.g., brain tissue), routine in pre-clinical work to procure samples for LRRK2 protein analysis, are unlikely to happen in clinical trials. However, phosphorylated and dimeric LRRK2 protein can be found within exosomes secreted into biofluids by a number of cell types (Fraser et al., 2013, 2016; Wang et al., 2017, 2019). Exosomes appear to have privileged access to tissue compartments across the body, including blood-brain barrier transparency, and represent heterogenous vesicles derived from many different cell types. Exosome-derived LRRK2 protein purified from biofluids like CSF, urine, saliva, and plasma, may provide a robust opportunity to predict and monitor LRRK2 inhibition across the body. However, the correlation between the LRRK2 changes observed in biofluids and those that occur in the brain (e.g., LRRK2 activity in neurons) will need extensive qualification with different therapeutic approaches in order to transition to an approved assay.

Routine blood collection from patients may also facilitate measurements of LRRK2 inhibition, in circulating cells where drug exposures are often much higher than in the brain. PBMCs can be isolated from whole blood and harbor abundant LRRK2 protein (Gardet et al., 2010; Kubo et al., 2010; Maekawa et al., 2010; Hakimi et al., 2011; Thévenet et al., 2011). *Ex vivo* treatment of PBMCs in culture with LRRK2 kinase inhibitors results in a reduction of constitutive LRRK2 phosphorylation without acute toxicity, as determined by phospho-Ser935 protein levels (Perera et al., 2016). However, short-duration *ex vivo* treatment (e.g., hours) may not recapitulate phenotypes associated with longer-duration treatment, like total LRRK2 protein reduction.

Urine represents another biofluid, collected without risk, that can be utilized to measure LRRK2 protein. Secreted LRRK2 in urinary exosomes is both dimerized and phosphorylated, proved to contain enzymatically active LRRK2 (Sen et al., 2009; Deng et al., 2011; Fraser et al., 2013). Urinary exosomes have been shown to contain elevated levels of autophosphorylated pS1292-LRRK2 and have utility in predicting LRRK2 mutation status and PD risk (Fraser et al., 2016; Wang et al., 2017). The collection of cerebrospinal fluid (CSF) is more invasive; however, it comes in direct contact with the brain and is routinely collected at least in early phase clinical trials. Neurons may be a major source of exosomes in the brain (Faure et al., 2006; Lachenal et al., 2011), although the exact source of LRRK2 protein in exosomes in CSF is not yet known. Like urinary exosomes, pS1292-LRRK2 can also be readily measured in exosomes isolated from CSF. Comparable amounts of total LRRK2 protein can be detected in CSF and urinary exosomes, but urinary exosomes exhibit lower pS1292-LRRK2 levels and more variability from sample to sample (Wang et al., 2017). Initial studies show that total LRRK2 protein and pS1292-LRRK2 levels in CSF and urine exosome fractions do not correlate within a subject (Wang et al., 2017), suggesting that there is cell specific regulation of LRRK2 expression and activity. There are no reports yet attempting to measure LRRK2 in saliva.

Our recent efforts in non-human primates treated with LRRK2 kinase inhibitors revealed that no single biomarker in any single biofluid is likely to detail the complexity of drug interactions across the body (Wang et al., 2020). The usage of informative panels of biomarkers, rather than reliance on an individual marker, is commonplace in fields with relatively mature validated biomarkers such as those used in acute kidney injury (Siew et al., 2011). As biomarker panels mature, the emphasis might shift from initial target engagement profiles toward association of responses with clinical outcomes. However, panels must be carefully contrived so that individual markers are not highly correlated with one another that might lead to over-fit and unhelpful models. Our experiences so-far in urine markers compared to CSF markers failed to detect any correlations within subjects (Wang et al., 2017), so panels utilizing different biofluids may be particularly efficacious in understanding drug effects.

## Initial Clinical Entry of LRRK2-Targeted Therapeutics

PK properties of small molecule LRRK2 kinase inhibitors have been refined over the last 10 years, demonstrating improved selectivity, brain permeability, and potency (Fell et al., 2015; Henderson et al., 2015; Scott et al., 2017; West, 2017; Kelly et al., 2018). Many of these molecules have already been evaluated in preclinical animal disease models to better understand the potential neuroprotection that could be afforded, as well as the extent of potentially adverse phenotypes, like those observed in LRRK2 knockout rodents (Daher et al., 2015; Fuji et al., 2015; Andersen et al., 2018; Kelly et al., 2018; Baptista et al., 2020). Antisense oligonucleotides (ASOs) have also recently emerged as a propitious strategy to treat multiple neurodegenerative diseases. ASOs are synthetic single-stranded nucleic acids that bind target mRNA, leading to the degradation of that target mRNA, and thereby reduce protein levels (Bennett et al., 2017). Importantly, intracerebral injections of ASOs allow for brain-specific targeting that is extensively distributed in cells and maintain a long duration of action (Kordasiewicz et al., 2012; Hung et al., 2013; Rigo et al., 2014). Several ASO therapeutics are already in clinical phase 1 trials for familial amyotrophic lateral sclerosis and Huntington’s disease, and Nusinersen has been approved by the FDA for the treatment of spinal muscular atrophy (ClinicalTrials.gov: NCT02623699, NCT02519036, and NCT02193074). Patient recruitment for phase 1 clinical trials of LRRK2 ASOs began June 2019 (ClinicalTrials.gov: NCT03976349). The use of LRRK2 ASOs aims to induce a long-term reduction in LRRK2 protein expression to reduce kinase activity as a therapeutic treatment.

With both small molecule LRRK2 kinase inhibitors and LRRK2-targeted ASOs, common biomarker platforms could be conceived to measure the reduction of total LRRK2 protein in CSF, and corresponding reductions of phospho-Rab substrates, caused by drug effects. Peripheral measures (e.g., blood and urine) would be less useful for establishing successful LRRK2 inhibition in the brain but could be useful in understanding inhibition profiles and dynamics of particular drugs. For example, early clinical trials may establish a strong correlation between plasma or urine LRRK2 inhibition biomarkers with those of CSF, obviating the need for CSF collection in larger populations in ongoing efficacy trials. Such a relationship appears to be emerging for both phospho-Tau protein and neurofilament light proteins, where CSF levels are highly correlated with plasma levels (Ashton et al., 2019; Forgrave et al., 2019).

## Concluding Remarks

Neurotherapeutics are considered to be at an inflection point as genetic understanding and disease mechanism continue to be elucidated (Ehlers, 2018). Several lines of evidence suggest biomarker driven approaches may be critical for the successful development of LRRK2-targeted therapeutics. Herein, we surveyed the pipeline for biomarker integration in the clinic and the most promising pharmacodynamic markers that might be considered for development. Measures will need to be sensitive, reproducible, and well-validated in different populations and laboratories (Figure 2). We further conclude that a single LRRK2-targeted biomarker will be insufficient to capture the complexity of LRRK2 inhibition biology across the body with any given drug. Rather, combinations of biomarkers would allow for a more holistic evaluation and better understanding of how different compounds affect LRRK2 throughout the body, and whether endpoints are achieved in the inhibition of LRRK2 in the brain. Combined efforts from academia, consortia, disease organizations, and biopharmaceutical companies will expedite the implementation of LRRK2-targeted biomarkers in drug development programs and clinical trials.

## Author Contributions

Both authors wrote and edited the manuscript. Both authors contributed to the article and approved the submitted version.

## Conflict of Interest

The authors declare that the research was conducted in the absence of any commercial or financial relationships that could be construed as a potential conflict of interest.

## References

[B1] Alegre-Abarrategui J. Christian H. Lufino M. M. P. Mutihac R. Venda L. L. Ansorge O. (2009). LRRK2 regulates autophagic activity and localizes to specific membrane microdomains in a novel human genomic reporter cellular model. *Hum. Mol. Genet.* 18 4022–4034. 10.1093/hmg/ddp346 19640926 PMC2758136

[B2] Altar C. A. Amakye D. Bounos D. Bloom J. Clack G. Dean R. (2008). A prototypical process for creating evidentiary standards for biomarkers and diagnostics. *Clin. Pharmacol. Ther.* 83 368–371. 10.1038/sj.clpt.6100451 18091762

[B3] Amur S. Lavange L. Zineh I. Buckman-Garner S. Woodcock J. (2015). Biomarker qualification: toward a multiple stakeholder framework for biomarker development, regulatory acceptance, and utilization. *Clin. Pharmacol. Ther.* 98 34–46. 10.1002/cpt.136 25868461

[B4] Andersen M. A. Wegener K. M. Larsen S. Badolo L. Smith G. P. Jeggo R. (2018). PFE-360-induced LRRK2 inhibition induces reversible, non-adverse renal changes in rats. *Toxicology* 395 15–22. 10.1016/j.tox.2018.01.003 29307545

[B5] Ashton N. J. Leuzy A. Lim Y. M. Troakes C. Hortobágyi T. Höglund K. (2019). Increased plasma neurofilament light chain concentration correlates with severity of post-mortem neurofibrillary tangle pathology and neurodegeneration. *Acta Neuropathol. Commun.* 7:5. 10.1186/s40478-018-0649-3 PMC6327431 30626432

[B6] Baptista M. A. S. Merchant K. Barrett T. Bhargava S. Bryce D. K. Ellis J. M. (2020). LRRK2 inhibitors induce reversible changes in nonhuman primate lungs without measurable pulmonary deficits. *Sci. Transl. Med.* 12:eaav0820. 10.1126/scitranslmed.aav0820 32321864

[B7] Bennett C. F. Baker B. F. Pham N. Swayze E. Geary R. S. (2017). Pharmacology of antisense drugs. *Annu. Rev. Pharm. Toxicol.* 57 81–105. 10.1146/annurev-pharmtox-010716-104846 27732800

[B8] Berg D. Holzmann C. Riess O. (2003). 14-3-3 proteins in the nervous system. *Nat. Rev. Neurosci.* 4:752. 10.1038/nrn1197 12951567

[B9] Biomarkers Definitions Working Group (2001). Biomarkers and surrogate endpoints: preferred definitions and conceptual framework. *Clin. Pharmacol. Ther.* 69 89–95. 10.1067/mcp.2001.113989 11240971

[B10] Biskup S. Moore D. J. Celsi F. Higashi S. West A. B. Andrabi S. A. (2006). Localization of LRRK2 to membranous and vesicular structures in mammalian brain. *Ann. Neurol.* 60 557–569. 10.1002/ana.21019 17120249

[B11] Biskup S. Moore D. J. Rea A. Lorenz-Deperieux B. Coombes C. E. Dawson V. L. (2007). Dynamic and redundant regulation of LRRK2 and LRRK1 expression. *BMC Neurosci.* 8:102. 10.1186/1471-2202-8-102 18045479 PMC2233633

[B12] Daher J. P. Abdelmotilib H. A. Hu X. Volpicelli-Daley L. A. Moehle M. S. Fraser K. B. (2015). Leucine-rich repeat kinase 2 (LRRK2) pharmacological inhibition abates alpha-synuclein gene-induced neurodegeneration. *J. Biol. Chem.* 290 19433–19444. 10.1074/jbc.m115.660001 26078453 PMC4528108

[B13] De Wit T. Baekelandt V. Lobbestael E. (2019). Inhibition of LRRK2 or casein kinase 1 results in LRRK2 protein destabilization. *Mol. Neurobiol.* 56 5273–5286. 10.1007/s12035-018-1449-2 30592011 PMC6657425

[B14] Delbroek L. Van Kolen K. Steegmans L. Da Cunha R. Mandemakers W. Daneels G. (2013). Development of an enzyme-linked immunosorbent assay for detection of cellular and in vivo LRRK2 S935 phosphorylation. *J. Pharm. Biomed. Anal.* 76 49–58. 10.1016/j.jpba.2012.12.002 23313773 PMC4196644

[B15] Deng X. Dzamko N. Prescott A. Davies P. Liu Q. Yang Q. (2011). Characterization of a selective inhibitor of the Parkinson’s disease kinase LRRK2. *Nat. Chem. Biol.* 7 203–205. 10.1038/nchembio.538 21378983 PMC3287420

[B16] Dodson M. W. Zhang T. Jiang C. Chen S. Guo M. (2012). Roles of the *Drosophila* LRRK2 homolog in Rab7-dependent lysosomal positioning. *Hum. Mol. Genet.* 21 1350–1363. 10.1093/hmg/ddr573 22171073 PMC3284123

[B17] Dowden H. Munro J. (2019). Trends in clinical success rates and therapeutic focus. *Nat. Rev. Drug Discov.* 18 495–496. 10.1038/d41573-019-00074-z 31267067

[B18] Dzamko N. Deak M. Hentati F. Reith A. D. Prescott A. R. Alessi D. R. (2010). Inhibition of LRRK2 kinase activity leads to dephosphorylation of Ser(910)/Ser(935), disruption of 14-3-3 binding and altered cytoplasmic localization. *Biochem. J.* 430 405–413. 10.1042/bj20100784 20659021 PMC3631100

[B19] Dzamko N. Inesta-Vaquera F. Zhang J. Xie C. Cai H. Arthur S. (2012). The IkappaB kinase family phosphorylates the Parkinson’s disease kinase LRRK2 at Ser935 and Ser910 during Toll-like receptor signaling. *PLoS One* 7:e39132. 10.1371/journal.pone.0039132 22723946 PMC3377608

[B20] Ehlers M. D. (2018). Neuroscience is the next oncology. *Innov. Clin. Neurosci.* 15 15–16. PMC5906084 29707421

[B21] Eyers P. A. (2018). Back to the future: new target-validated Rab antibodies for evaluating LRRK2 signalling in cell biology and Parkinson’s disease. *Biochem. J.* 475 185–189. 10.1042/bcj20170870 29305429 PMC5754967

[B22] Faure J. Lachenal G. Court M. Hirrlinger J. Chatellard-Causse C. Blot B. (2006). Exosomes are released by cultured cortical neurones. *Mol. Cell. Neurosci.* 31 642–648. 10.1016/j.mcn.2005.12.003 16446100

[B23] Fell M. J. Mirescu C. Basu K. Cheewatrakoolpong B. Demong D. E. Ellis J. M. (2015). MLi-2, a potent, selective, and centrally active compound for exploring the therapeutic potential and safety of LRRK2 Kinase inhibition. *J. Pharmacol. Exp. Ther.* 355 397–409. 10.1124/jpet.115.227587 26407721

[B24] Forgrave L. M. Ma M. Best J. R. Demarco M. L. (2019). The diagnostic performance of neurofilament light chain in CSF and blood for Alzheimer’s disease, frontotemporal dementia, and amyotrophic lateral sclerosis: a systematic review and meta-analysis. *Alzheimer’s Dement.* 11 730–743. 10.1016/j.dadm.2019.08.009 31909174 PMC6939029

[B25] Fraser K. B. Moehle M. S. Alcalay R. N. West A. B. (2016). Urinary LRRK2 phosphorylation predicts parkinsonian phenotypes in G2019S LRRK2 carriers. *Neurology* 86 994–999. 10.1212/wnl.0000000000002436 26865512 PMC4799717

[B26] Fraser K. B. Moehle M. S. Daher J. P. Webber P. J. Williams J. Y. Stewart C. A. (2013). LRRK2 secretion in exosomes is regulated by 14-3-3. *Hum. Mol. Genet.* 22 4988–5000. 10.1093/hmg/ddt346 23886663 PMC3836478

[B27] Fuji R. N. Flagella M. Baca M. Baptista M. A. Brodbeck J. Chan B. K. (2015). Effect of selective LRRK2 kinase inhibition on nonhuman primate lung. *Sci. Transl. Med.* 7:273ra215. 10.1126/scitranslmed.aaa3634 25653221

[B28] Gardet A. Benita Y. Li C. Sands B. E. Ballester I. Stevens C. (2010). LRRK2 is involved in the IFN-gamma response and host response to pathogens. *J. Immunol.* 185 5577–5585. 10.4049/jimmunol.1000548 20921534 PMC3156100

[B29] Giesert F. Glasl L. Zimprich A. Ernst L. Piccoli G. Stautner C. (2017). The pathogenic LRRK2 R1441C mutation induces specific deficits modeling the prodromal phase of Parkinson’s disease in the mouse. *Neurobiol. Dis.* 105 179–193. 10.1016/j.nbd.2017.05.013 28576705

[B30] Gloeckner C. J. Boldt K. Von Zweydorf F. Helm S. Wiesent L. Sarioglu H. (2010). Phosphopeptide analysis reveals two discrete clusters of phosphorylation in the N-terminus and the Roc domain of the Parkinson-disease associated protein kinase LRRK2. *J. Proteome Res.* 9 1738–1745. 10.1021/pr9008578 20108944

[B31] Goodsaid F. Papaluca M. (2010). Evolution of biomarker qualification at the health authorities. *Nat. Biotechnol.* 28 441–443. 10.1038/nbt0510-441 20458312

[B32] Greggio E. Taymans J. M. Zhen E. Y. Ryder J. Vancraenenbroeck R. Beilina A. (2009). The Parkinson’s disease kinase LRRK2 autophosphorylates its GTPase domain at multiple sites. *Biochem. Biophys. Res. Commun.* 389 449–454. 10.1016/j.bbrc.2009.08.163 19733152 PMC2759846

[B33] Hakimi M. Selvanantham T. Swinton E. Padmore R. F. Tong Y. Kabbach G. (2011). Parkinson’s disease-linked LRRK2 is expressed in circulating and tissue immune cells and upregulated following recognition of microbial structures. *J. Neural Transm.* 118 795–808. 10.1007/s00702-011-0653-2 21552986 PMC3376651

[B34] Hatano T. Kubo S.-I. Imai S. Maeda M. Ishikawa K. Mizuno Y. (2007). Leucine-rich repeat kinase 2 associates with lipid rafts. *Hum. Mol. Genet.* 16 678–690. 10.1093/hmg/ddm013 17341485

[B35] Healy D. G. Falchi M. O’sullivan S. S. Bonifati V. Durr A. Bressman S. (2008). Phenotype, genotype, and worldwide genetic penetrance of LRRK2-associated Parkinson’s disease: a case-control study. *Lancet Neurol.* 7 583–590. 10.1016/s1474-4422(08)70117-0 18539534 PMC2832754

[B36] Henderson J. L. Kormos B. L. Hayward M. M. Coffman K. J. Jasti J. Kurumbail R. G. (2015). Discovery and preclinical profiling of 3-[4-(morpholin-4-yl)-7H-pyrrolo[2,3-d]pyrimidin-5-yl]benzonitrile (PF-06447475), a highly potent, selective, brain penetrant, and in vivo active LRRK2 kinase inhibitor. *J. Med. Chem.* 58 419–432. 10.1021/jm5014055 25353650

[B37] Herzig M. C. Kolly C. Persohn E. Theil D. Schweizer T. Hafner T. (2011). LRRK2 protein levels are determined by kinase function and are crucial for kidney and lung homeostasis in mice. *Hum. Mol. Genet.* 20 4209–4223. 10.1093/hmg/ddr348 21828077 PMC3188995

[B38] Higashi S. Moore D. J. Yamamoto R. Minegishi M. Sato K. Togo T. (2009). Abnormal localization of leucine-rich repeat kinase 2 to the endosomal-lysosomal compartment in lewy body disease. *J. Neuropathol. Exp. Neurol.* 68 994–1005. 10.1097/nen.0b013e3181b44ed8 19680143 PMC2768772

[B39] Hopkins A. L. Groom C. R. (2002). The druggable genome. *Nat. Rev. Drug Discov.* 1 727–730. 12209152 10.1038/nrd892

[B40] Hung G. Xiao X. Peralta R. Bhattacharjee G. Murray S. Norris D. (2013). Characterization of target mRNA reduction through in situ RNA hybridization in multiple organ systems following systemic antisense treatment in animals. *Nucleic Acid Ther.* 23 369–378. 10.1089/nat.2013.0443 24161045

[B41] Ito G. Katsemonova K. Tonelli F. Lis P. Baptista M. A. S. Shpiro N. (2016). Phos-tag analysis of Rab10 phosphorylation by LRRK2: a powerful assay for assessing kinase function and inhibitors. *Biochem. J.* 473 2671–2685. 10.1042/bcj20160557 27474410 PMC5003698

[B42] Kelly K. Wang S. Boddu R. Liu Z. Moukha-Chafiq O. Augelli-Szafran C. (2018). The G2019S mutation in LRRK2 imparts resiliency to kinase inhibition. *Exp. Neurol.* 309 1–13. 10.1016/j.expneurol.2018.07.012 30048714 PMC7041630

[B43] Kordasiewicz H. B. Stanek L. M. Wancewicz E. V. Mazur C. Mcalonis M. M. Pytel K. A. (2012). Sustained therapeutic reversal of Huntington’s disease by transient repression of huntingtin synthesis. *Neuron* 74 1031–1044. 10.1016/j.neuron.2012.05.009 22726834 PMC3383626

[B44] Kubo M. Kamiya Y. Nagashima R. Maekawa T. Eshima K. Azuma S. (2010). LRRK2 is expressed in B-2 but not in B-1 B cells, and downregulated by cellular activation. *J. Neuroimmunol.* 229 123–128. 10.1016/j.jneuroim.2010.07.021 20728949

[B45] Lachenal G. Pernet-Gallay K. Chivet M. Hemming F. J. Belly A. Bodon G. (2011). Release of exosomes from differentiated neurons and its regulation by synaptic glutamatergic activity. *Mol. Cell. Neurosci.* 46 409–418. 10.1016/j.mcn.2010.11.004 21111824

[B46] Lavalley N. J. Slone S. R. Ding H. West A. B. Yacoubian T. A. (2016). 14-3-3 Proteins regulate mutant LRRK2 kinase activity and neurite shortening. *Hum. Mol. Genet.* 25 109–122. 10.1093/hmg/ddv453 26546614 PMC4690493

[B47] Li X. Wang Q. J. Pan N. Lee S. Zhao Y. Chait B. T. (2011). Phosphorylation-dependent 14-3-3 Binding to LRRK2 is impaired by common mutations of familial Parkinson’s disease. *PLoS One* 6:e17153. 10.1371/journal.pone.0017153 21390248 PMC3046972

[B48] Liu Z. Galemmo R. A. Jr. Fraser K. B. Moehle M. S. Sen S. Volpicelli-Daley L. A. (2014). Unique functional and structural properties of the LRRK2 protein ATP-binding pocket. *J. Biol. Chem.* 289 32937–32951. 10.1074/jbc.m114.602318 25228699 PMC4239640

[B49] Liu Z. Mobley J. A. Delucas L. J. Kahn R. A. West A. B. (2016). LRRK2 autophosphorylation enhances its GTPase activity. *FASEB J.* 30 336–347. 10.1096/fj.15-277095 26396237 PMC4684519

[B50] Lobbestael E. Civiero L. De Wit T. Taymans J. M. Greggio E. Baekelandt V. (2016). Pharmacological LRRK2 kinase inhibition induces LRRK2 protein destabilization and proteasomal degradation. *Sci. Rep.* 6:33897. 10.1038/srep33897 PMC5034242 27658356

[B51] Lobbestael E. Zhao J. Rudenko I. N. Beylina A. Gao F. Wetter J. (2013). Identification of protein phosphatase 1 as a regulator of the LRRK2 phosphorylation cycle. *Biochem. J.* 456 119–128. 10.1042/bj20121772 23937259 PMC5141581

[B52] Lopes G. Parker J. L. Willan A. Shah S. Weerasinghe A. Rubinger D. (2015). The role of biomarkers in improving clinical trial success: a study of 1,079 oncology drugs. *J. Clin. Oncol.* 33:e17804 10.1200/jco.2015.33.15_suppl.e17804

[B53] Mabrouk O. S. Chen S. Edwards A. L. Yang M. Hirst W. D. Graham D. L. (2020). Quantitative measurements of LRRK2 in human cerebrospinal fluid demonstrates increased levels in G2019S Patients. *Front. Neurosci.* 14:526. 10.3389/fnins.2020.00526 32523511 PMC7262382

[B54] Maekawa T. Kubo M. Yokoyama I. Ohta E. Obata F. (2010). Age-dependent and cell-population-restricted LRRK2 expression in normal mouse spleen. *Biochem. Biophys. Res. Commun.* 392 431–435. 10.1016/j.bbrc.2010.01.041 20079710

[B55] Matta S. Van Kolen K. da Cunha R. Van den bogaart G. Mandemakers W. Miskiewicz K. (2012). LRRK2 controls an EndoA phosphorylation cycle in synaptic endocytosis. *Neuron* 75 1008–1021. 10.1016/j.neuron.2012.08.022 22998870

[B56] Morgan P. Brown D. G. Lennard S. Anderton M. J. Barrett J. C. Eriksson U. (2018). Impact of a five-dimensional framework on R&D productivity at AstraZeneca. *Nat. Rev. Drug Discov.* 17 167–181. 29348681 10.1038/nrd.2017.244

[B57] Morgan P. Van Der Graaf P. H. Arrowsmith J. Feltner D. E. Drummond K. S. Wegner C. D. (2012). Can the flow of medicines be improved? Fundamental pharmacokinetic and pharmacological principles toward improving Phase II survival. *Drug Discov. Today* 17 419–424. 10.1016/j.drudis.2011.12.020 22227532

[B58] Muda K. Bertinetti D. Gesellchen F. Hermann J. S. Von Zweydorf F. Geerlof A. (2014). Parkinson-related LRRK2 mutation R1441C/G/H impairs PKA phosphorylation of LRRK2 and disrupts its interaction with 14-3-3. *Proc. Natl. Acad. Sci. U.S.A.* 111 E34–E43. 24351927 10.1073/pnas.1312701111 PMC3890784

[B59] Nichols R. J. Dzamko N. Morrice N. A. Campbell D. G. Deak M. Ordureau A. (2010). 14-3-3 binding to LRRK2 is disrupted by multiple Parkinson’s disease-associated mutations and regulates cytoplasmic localization. *Biochem. J.* 430 393–404. 10.1042/bj20100483 20642453 PMC2932554

[B60] Padmanabhan S. Lanz T. A. Gorman D. Wolfe M. Joyce A. Cabrera C. (2020). An assessment of LRRK2 serine 935 phosphorylation in human peripheral blood mononuclear cells in idiopathic Parkinson’s Disease and G2019S *LRRK2* cohorts. *J. Parkinsons Dis.* 10, 623–629. 10.3233/JPD-191786 32007961 PMC7242833

[B61] Paweletz C. P. Andersen J. N. Pollock R. Nagashima K. Hayashi M. L. Yu S. U. (2011). Identification of direct target engagement biomarkers for kinase-targeted therapeutics. *PLoS One* 6:e26459. 10.1371/journal.pone.0026459 22039492 PMC3200335

[B62] Perera G. Ranola M. Rowe D. B. Halliday G. M. Dzamko N. (2016). Inhibitor treatment of peripheral mononuclear cells from Parkinson’s disease patients further validates LRRK2 dephosphorylation as a pharmacodynamic biomarker. *Sci. Rep.* 6:31391. 10.1038/srep31391 PMC4977566 27503089

[B63] Piccoli G. Condliffe S. B. Bauer M. Giesert F. Boldt K. De Astis S. (2011). LRRK2 controls synaptic vesicle storage and mobilization within the recycling pool. *J. Neurosci.* 31:2225. 10.1523/jneurosci.3730-10.2011 21307259 PMC6633036

[B64] Pungaliya P. P. Bai Y. Lipinski K. Anand V. S. Sen S. Brown E. L. (2010). Identification and characterization of a leucine-rich repeat kinase 2 (LRRK2) consensus phosphorylation motif. *PLoS One* 5:e13672. 10.1371/journal.pone.0013672 21060682 PMC2965117

[B65] Rigo F. Chun S. J. Norris D. A. Hung G. Lee S. Matson J. (2014). Pharmacology of a central nervous system delivered 2’-O-methoxyethyl–modified survival of motor neuron splicing oligonucleotide in mice and nonhuman primates. *J. Pharmacol. Exp. Ther.* 350 46–55. 10.1124/jpet.113.212407 24784568 PMC4056267

[B66] Scott J. D. Demong D. E. Greshock T. J. Basu K. Dai X. Harris J. (2017). Discovery of a 3-(4-Pyrimidinyl) indazole (MLi-2), an orally available and selective leucine-rich repeat kinase 2 (LRRK2) inhibitor that reduces brain kinase activity. *J. Med. Chem.* 60 2983–2992. 28245354 10.1021/acs.jmedchem.7b00045

[B67] Sen S. Webber P. J. West A. B. (2009). Dependence of leucine-rich repeat kinase 2 (LRRK2) kinase activity on dimerization. *J. Biol. Chem.* 284 36346–36356. 10.1074/jbc.m109.025437 19826009 PMC2794750

[B68] Sheng Z. Zhang S. Bustos D. Kleinheinz T. Le Pichon C. E. Dominguez S. L. (2012). Ser1292 autophosphorylation is an indicator of LRRK2 kinase activity and contributes to the cellular effects of PD mutations. *Sci. Transl. Med.* 4:164ra161. 10.1126/scitranslmed.3004485 23241745

[B69] Siew E. D. Ware L. B. Ikizler T. A. (2011). Biological markers of acute kidney injury. *J. Am. Soc. Nephrol.* 22 810–820. 21493774 10.1681/ASN.2010080796

[B70] Smietana K. Siatkowski M. Møller M. (2016). Trends in clinical success rates. *Nat. Rev. Drug Discov.* 15 379–380. 10.1038/nrd.2016.85 27199245

[B71] Steger M. Tonelli F. Ito G. Davies P. Trost M. Vetter M. (2016). Phosphoproteomics reveals that Parkinson’s disease kinase LRRK2 regulates a subset of Rab GTPases. *eLife* 5:e12813. 10.7554/eLife.12813 PMC4769169 26824392

[B72] Thévenet J. Pescini Gobert R. van Huijsduijnen R. H. Wiessner C. Sagot Y. J. (2011). Regulation of LRRK2 expression points to a functional role in human monocyte maturation. *PLoS One* 6:e21519. 10.1371/journal.pone.0021519 21738687 PMC3124520

[B73] Thirstrup K. Dachsel J. C. Oppermann F. S. Williamson D. S. Smith G. P. Fog K. (2017). Selective LRRK2 kinase inhibition reduces phosphorylation of endogenous Rab10 and Rab12 in human peripheral mononuclear blood cells. *Sci. Rep.* 7:10300. 10.1038/s41598-017-10501-z PMC5578959 28860483

[B74] Tong Y. Yamaguchi H. Giaime E. Boyle S. Kopan R. Kelleher R. J. III (2010). Loss of leucine-rich repeat kinase 2 causes impairment of protein degradation pathways, accumulation of alpha-synuclein, and apoptotic cell death in aged mice. *Proc. Natl. Acad. Sci. U.S.A.* 107 9879–9884. 10.1073/pnas.1004676107 20457918 PMC2906862

[B75] Wang S. Kelly K. Schussler N. Boularand S. Dubois L. Hsieh F. (2020). Predictive markers of LRRK2 inhibition in biofluids. *bioRxiv* [Preprint] 10.1101/2020.01.28.923557

[B76] Wang S. Kojima K. Mobley J. A. West A. B. (2019). Proteomic analysis of urinary extracellular vesicles reveal biomarkers for neurologic disease. *EBioMedicine* 45 351–361. 10.1016/j.ebiom.2019.06.021 31229437 PMC6642358

[B77] Wang S. Liu Z. Ye T. Mabrouk O. S. Maltbie T. Aasly J. (2017). Elevated LRRK2 autophosphorylation in brain-derived and peripheral exosomes in LRRK2 mutation carriers. *Acta Neuropathol. Commun.* 5:86. 10.1186/s40478-017-0492-y PMC5700679 29166931

[B78] Webber P. J. Smith A. D. Sen S. Renfrow M. B. Mobley J. A. West A. B. (2011). Autophosphorylation in the leucine-rich repeat kinase 2 (LRRK2) GTPase domain modifies kinase and GTP-binding activities. *J. Mol. Biol.* 412 94–110. 10.1016/j.jmb.2011.07.033 21806997 PMC3158845

[B79] West A. B. (2015). Ten years and counting: moving leucine-rich repeat kinase 2 inhibitors to the clinic. *Mov. Disord.* 30 180–189. 10.1002/mds.26075 25448543 PMC4318704

[B80] West A. B. (2017). Achieving neuroprotection with LRRK2 kinase inhibitors in Parkinson disease. *Exp. Neurol.* 298 236–245. 10.1016/j.expneurol.2017.07.019 28764903 PMC5693612

[B81] West A. B. Moore D. J. Biskup S. Bugayenko A. Smith W. W. Ross C. A. (2005). Parkinson’s disease-associated mutations in leucine-rich repeat kinase 2 augment kinase activity. *Proc. Natl. Acad. Sci. U.S.A.* 102 16842–16847. 10.1073/pnas.0507360102 16269541 PMC1283829

[B82] West A. B. Moore D. J. Choi C. Andrabi S. A. Li X. Dikeman D. (2007). Parkinson’s disease-associated mutations in LRRK2 link enhanced GTP-binding and kinase activities to neuronal toxicity. *Hum. Mol. Genet.* 16 223–232. 10.1093/hmg/ddl471 17200152

[B83] Willms E. Cabañas C. Mäger I. Wood M. J. A. Vader P. (2018). Extracellular vesicle heterogeneity: subpopulations, isolation techniques, and diverse functions in cancer progression. *Front. Immunol.* 9:738. 10.3389/fimmu.2018.00738 29760691 PMC5936763

[B84] Wishart D. S. Knox C. Guo A. C. Shrivastava S. Hassanali M. Stothard P. (2006). DrugBank: a comprehensive resource for in silico drug discovery and exploration. *Nucleic Acids Res.* 34 D668–D672. 16381955 10.1093/nar/gkj067 PMC1347430

[B85] Wong C. H. Siah K. W. Lo A. W. (2019). Estimation of clinical trial success rates and related parameters. *Biostatistics* 20 273–286. 10.1093/biostatistics/kxx069 29394327 PMC6409418

[B86] Woodcock J. Woosley R. (2008). The FDA critical path initiative and its influence on new drug development. *Annu. Rev. Med.* 59 1–12. 10.1146/annurev.med.59.090506.155819 18186700

[B87] Wu X. Liu X. Koul S. Lee C. Y. Zhang Z. Halmos B. (2014). AXL kinase as a novel target for cancer therapy. *Oncotarget* 5 9546–9563. 10.18632/oncotarget.2542 25337673 PMC4259419

[B88] Zhang Y. Du Z. Zhang M. (2016). Biomarker development in MET-targeted therapy. *Oncotarget* 7 37370–37389. 10.18632/oncotarget.8276 27013592 PMC5095083

[B89] Zhao J. Molitor T. P. Langston J. W. Nichols R. J. (2015). LRRK2 dephosphorylation increases its ubiquitination. *Biochem. J.* 469 107–120. 10.1042/bj20141305 25939886 PMC4613513

[B90] Zimprich A. Biskup S. Leitner P. Lichtner P. Farrer M. Lincoln S. (2004). Mutations in LRRK2 cause autosomal-dominant parkinsonism with pleomorphic pathology. *Neuron* 44 601–607. 10.1016/j.neuron.2004.11.005 15541309

